# Glucoraphanin Accumulation via Glucoraphanin Synthesis Promotion during Broccoli Germination

**DOI:** 10.3390/foods13010041

**Published:** 2023-12-21

**Authors:** Guangmin Liu, Hongju He, Pengjie Wang, Xirui Zhao, Fazheng Ren

**Affiliations:** 1 Key Laboratory of Functional Dairy, Ministry of Education, Department of Nutrition and Health, China Agricultural University, Beijing 100083, China; liuguangmin@iapn.org.cn (G.L.); wpj@cau.edu.cn (P.W.); 2 Institute of Agri-Food Processing and Nutrition, Beijing Academy of Agricultural and Forestry Sciences, Beijing 100097, China; hehongju@iapn.org.cn (H.H.); zhaoxirui@iapn.org.cn (X.Z.)

**Keywords:** glucoraphanin, biosynthesis, gene expression, broccoli seedling

## Abstract

Glucoraphanin is an important glucosinolate which is widely distributed in Brassica vegetables and poses an anticancer effect to humans. Although researchers have paid a lot of attention to the changes in glucoraphanin concentration in seedlings of broccoli over 1–2 weeks, there has been little research focusing on the total whole-sprout glucoraphanin content within broccoli seedlings over 1–5 weeks. However, it is necessary to clarify the changes in total glucoraphanin content during the broccoli sprouting stage as broccoli seedlings are novel plant foods. This research explored glucoraphanin absolute accumulation and the biosynthesis mechanism in broccoli seedlings during a 5-week growth period. The results showed that glucoraphanin accumulation content was higher at week 4 than in the seeds. Moreover, the relative DL-methionine contents increased significantly after 3 weeks. Glucoraphanin synthetic gene expression levels were increased after 3 weeks, but the gene expressions of *AOP3* (encoding 2-oxoglutarate-dependent dioxygenases) and *MYR* (encoding myrosinase) were significantly decreased. Furthermore, the 20 essential DEGs obtained can provide new insight into understanding the developmental regulation of broccoli seedlings. In addition, the results can also provide information on how to obtain higher glucoraphanin contents in broccoli sprouts.

## 1. Introduction

Glucoraphanin is a glucosinolate that is widely found in Brassica vegetables and is frequently consumed by people worldwide [1,2]. Analyses of vegetables and fruits have revealed that broccoli has the highest glucoraphanin content [3]. Glucosinolates (GSLs), which are a class of nitrogen-containing and sulfur-containing compounds, consist of a glycosidic group, a sulfonate aldoxime group, and an R group [4,5]. According to the synthetic pathway, glucosinolate can be divided into aliphatic glucosinolate, benzenic glucosinolate, and indole glucosinolate [6]. Thus, based on its structure, glucoraphanin can be considered an aliphatic glucosinolate [7]. Research has shown that glucoraphanin can be degraded to sulforaphane by myrosinase (MYR; e.g., TGG1 and TGG2) after the breakdown of broccoli and can further impact human health [8,9,10]. For example, the regular consumption of broccoli may provide some protection against COVID-19 as glucoraphanin can be degraded to sulforaphane to act against viral replication in vivo [11]. Moreover, the metabolite of glucoraphanin has anti-angiogenesis, anti-metastasis and anti-inflammatory functions. Additionally, it has been revealed to inhibit the proliferation and promote the apoptosis of cancer cells [12]. Overall, human health may be improved by increasing the consumption of Brassica vegetables due to the beneficial effects of glucoraphanin. Researchers worldwide have focused on the glucoraphanin biosynthetic pathway in order to maximize the glucoraphanin content in mature Brassica plants or Brassica seedlings.

Researchers determined that glucoraphanin biosynthesis involves three main processes. Firstly, the glucoraphanin side chain is extended. In the first stage, DL-methionine and the synthesis of 2-oxo acids via S-adenosyl methionine (SAMe) are involved [13,14]. Secondly, the core structure is synthesized via a five-step process (oxidation, conjugation, carbon-sulfur (C-S) cleavage, glycosylation, and sulfation) [15]. During these five steps, key regulatory genes (SUR1, CYP79F1, and GYP83A1) and genes encoding enzymes, such as C-S lyase (for C-S cleavage) and sulfotransferases (SOT17 and SOT18 involved in sulfation), are the most important factors restricting the synthesis of the core structure [16,17]. Thirdly, the side chain is modified in a process controlled by a flavin-containing monooxygenase (FMOGS-OX), which mediates the formation of different glucosinolate structures [18]. In addition, AOP3 (2-oxoglutarate-dependent dioxygenases) can catalyze the conversion of glucoraphanin to hydroxyalkyl glucosinolate, whereas MYR (myrosinase) can catalyze the degradation of glucoraphanin to sulforaphane in all Brassicaceae [19,20].

Following the gradual characterization and clarification of the biological functions of glucoraphanin and its metabolites, researchers have focused on the regulation of glucoraphanin biosynthesis [21]. Almuhayawi et al. demonstrated that improving quinone reductase and glutathione S-transferase activities can increase glucoraphanin and glucosinolate accumulation in broccoli [22]. Inorganic salt ions also affect the accumulation of glucoraphanin in broccoli seeds. Specifically, calcium ions promote the accumulation of glucoraphanin by inhibiting MYR activity, while selenium ions regulate the expression of sulfate transporter genes and several cytochrome P450 genes [23,24]. Researchers also determined that exposure to light-emitting diode (LED) light, especially blue and red-blue LED lights, can induce the expression of aliphatic glucosinolate biosynthetic genes (CYP79F1, CYP83A1, and UGT74B1) [15,25,26].

However, researchers have primarily focused on the changes in glucosinolate concentration (on a per gram basis) in seedlings of broccoli over 1–2 weeks. Unfortunately, there has been no research on the changes in the total glucoraphanin content during the germination process (1–5 week) of broccoli (in a whole sprout). However, researchers have determined that broccoli seedlings are novel plant foods because of their rich bioactive compound composition compared to maturation plants, such as glucosinolates [27]. Additionally, there are high glucosinolate levels in broccoli seedlings (10–100 times higher than in maturation tissues) during this stage of growth [28], and so glucoraphanin and its metabolite are nutritional compounds. Thus, the glucoraphanin biosynthesis pathway and its total content changes in the whole sprouts during broccoli germination should be elucidated.

This research aimed to explore the factors regulating the synthesis and degradation of glucoraphanin during broccoli seedling development. The study data may be used to produce broccoli seedlings with high glucoraphanin contents, thereby increasing the nutritional value of broccoli seedlings. The findings of this study may also be applicable to other Brassica sprout vegetables.

## 2. Materials and Methods

### 2.1. Materials

Broccoli seeds (BHL106) were obtained from the Vegetable Research Center of the Beijing Academy of Agricultural and Forestry Sciences. Methanol, formic acid, ammonium acetate, acetonitrile, Sinigrin (SIN), Gluconapin (NAP), Progoitrin (PRO), Glucotropaeolin (TRO), Glucoerucin (ERU), Gluconasturtiin (NAS), Glucoraphenin (RAE), Glucobrassicin (GBC), Glucoalyssin (ALY), 4-Methoxyglucobrassicin (4ME), Neoglucobrassicin (NEO), and glucoraphanin were purchased from PhytoLab GmbH & Co. KG (Vestenbergsgreuth, Middle Franconia, Germany). The purity of all chemical substances was >99%. Individual stock solutions of the 13 GSLs were prepared in 70% methanol. Ultrapure deionized water (18.2 MΩ) was prepared using Milli-Q purification systems (Millipore, MA, USA).

### 2.2. Experimental Design and Sample Collection

Broccoli seeds (BHL106) were immersed in deionized water for 3 h and then germinated in seedling trays filled with growing or supporting medium (peat/vermiculite/perlite; 2:1:1). The resulting seedlings were cultured at 25 °C (day) and 18 °C (night) with a 12 h photoperiod (200 μmol m^−2^ s^−1^ light intensity) and 75% relative humidity. In addition to the seeds (*n* = 6, W0 group), the edible plant parts were collected at 1 week (*n* = 6, W1 group), 2 weeks (*n* = 6, W2 group), 3 weeks (*n* = 6, W3 group), 4 weeks (*n* = 6, W4 group), and 5 weeks (*n* = 6, W5 group) after germination. The length and fresh weight of the collected broccoli seedlings in each group were measured. The seeds (W0 group) were left with no seeding time. All samples were frozen immediately and kept at −80 °C until analysis.

### 2.3. Analysis of Glucosinolate and Glucoraphanin Accumulation

The detection method of glucosinolate was based on Yu et al. [29]. For the frozen-fresh sample powder, 1.00 g of sample was placed in a 50 mL centrifuge tube and treated with 10 mL of 80% methanol (*v*/*v*) containing 1000 ng/mL glucotropaeolin (TRO) as an internal standard. The extraction method consisted of incubation at 75 °C for 20 min and sonication for 20 min at room temperature. Subsequently, the extracts were centrifuged at 3000× *g* for 15 min, with 1 mL of the supernatant diluted with ultrapure water to 10 mL and filtered through a 0.22 um nylon syringe filter for UHPLC-MS/MS analysis.

Ultra-high-performance liquid chromatography (UHPLC) and tandem mass spectrometry (MS/MS) analyses were performed using a Waters Acquity I-Class system and a Xevo TQ-S micro triple quadrupole mass spectrometer. Samples were injected into a Waters Acquity UPLC^®^ BEH C18 column (100 × 2.1 mm, 1.7 µm particle size) maintained at 30 °C using a 12 min linear gradient and a flow rate of 0.2 mL/min. The mobile phases were eluent A (0.1% formic acid in water) and eluent B (100% methanol). The gradient elution was as follows: 0 min, 90% A; 1.0 min, 90% A; 3.0 min, 75% A; 5.0 min, 40% A; 6.0 min, 0% A; 6.2 min, 90% A; and 9.0 min, 90% A.

The glucoraphanin accumulation ratio during broccoli seedling development was calculated using the following formula: total glucoraphanin content in seedlings/total glucoraphanin content in seeds.

### 2.4. Measurement of Glucoraphanin Biosynthesis Pathway Metabolites

Tissues (100 mg) were ground in liquid nitrogen and the homogenate was resuspended in prechilled 80% methanol and thoroughly vortexed. The samples were incubated on ice for 5 min and then centrifuged for 20 min (15,000× *g*, 4 °C). An aliquot of the supernatant was diluted with LC-MS-grade water for a final methanol concentration of 53%. The samples were subsequently transferred to a fresh Eppendorf tube and centrifuged for 20 min at 15,000× *g* and 4 °C. Finally, the supernatant was collected for the UHPLC-MS/MS analysis.

Metabolites were detected according to a previously described method that was modified slightly [30]. The UHPLC-MS/MS analysis was performed by Novogene Co., Ltd. (Beijing, China) using the Vanquish UHPLC system (Thermo Fisher, Karlsruhe, Germany) coupled with an Orbitrap Q Exactive™ HF-X mass spectrometer (Thermo Fisher). Samples were injected into the Hypersil Gold column (100 × 2.1 mm, 1.9 μm) using a 12 min linear gradient and a flow rate of 0.2 mL/min. The mobile phases for the positive polarity mode were eluent A (0.1% formic acid in water) and eluent B (methanol), whereas the mobile phases for the negative polarity mode were eluent A (5 mM ammonium acetate, pH 9.0) and eluent B (methanol). The gradient elution was as follows: 2% B, 1.5 min; 2–85% B, 3 min; 85–100% B, 10 min; 100–2% B, 10.1 min; and 2% B, 12 min. The Q Exactive™ HF-X mass spectrometer was operated in the positive/negative polarity modes, with a spray voltage of 3.5 kV, capillary temperature of 320 °C, sheath gas flow rate of 35 psi, auxiliary gas flow rate of 10 L/min, S-lens RF level of 60, and auxiliary gas heater temperature of 350 °C.

The metabolites were annotated using the following databases: KEGG (https://www.genome.jp/kegg/pathway.html, accessed on 30 November 2023), HMDB (https://hmdb.ca/metabolites, accessed on 30 November 2023), and LIPID Maps (http://www.lipidmaps.org/, accessed on 30 November 2023). We performed a univariate analysis (*t*-test) to calculate the statistical significance (*p*-value). The following criteria were used to identify differentially abundant metabolites: VIP > 1, *p* < 0.05, and fold-change ≥2 or ≤0.5.

### 2.5. Analysis of Glucoraphanin Synthesis-Related Gene Expression

The RNA integrity was assessed using the RNA Nano 6000 Assay Kit and the 2100 Bioanalyzer (Agilent Technologies, California, CA, USA).

Total RNA was used for the construction of transcriptome sequencing libraries. Briefly, mRNA was purified from the total RNA using oligo-d(T) magnetic beads. The mRNA was fragmented at high temperatures using divalent cations and the First Strand Synthesis Reaction Buffer (5×). First-strand cDNA was synthesized using a random hexamer primer and M-MuLV Reverse Transcriptase (RNase H-). The second cDNA strand was synthesized using DNA Polymerase I and RNase H. The overhanging ends were converted to blunt ends via exonuclease/polymerase activities. After adenylating the 3′ end of the cDNA fragments, an adapter with a hairpin loop structure was ligated prior to the hybridization. In order to select cDNA fragments with the preferred length (370–420 bp), the library fragments were purified using the AMPure XP system (Beckman Coulter, Beverly, MA, USA). The size-selected fragments were amplified via PCR using Phusion High-Fidelity DNA polymerase, universal PCR primers, and an index (X) primer. The PCR products were purified using the AMPure XP system, after which the library quality was assessed using the 2100 Bioanalyzer.

The clustering of the index-coded samples was performed using the cBot Cluster Generation System and the TruSeq PE Cluster Kit (v3-cBot-HS) (Illumina, San Diego, CA, USA). After generating clusters, the libraries were sequenced on the Illumina NovaSeq platform to generate 150 bp paired-end reads.

Feature Counts (v1.5.0-p3) was used to count the reads mapped to each gene. The FPKM value (i.e., expected number of fragments per kilobase of transcript sequence per million base pairs sequenced) for each gene was calculated according to the gene length and the number of reads mapped to the gene. Because the FPKM value reflects the effect of the sequencing depth and gene length on the read count, it is commonly used to estimate gene expression levels.

Differentially expressed genes (DEGs) between two conditions/groups (two biological replicates per condition) were identified using the DESeq2 R package (1.20.0). Briefly, DESeq2 provides statistical routines for determining differential expression using digital gene expression data and a model based on negative binomial distribution. The resulting *p*-values were adjusted using Benjamini and Hochberg’s approach for controlling the false discovery rate. Genes with an adjusted *p* ≤ 0.05 revealed via DESeq2 were designated as DEGs.

### 2.6. RNA Isolation and Quantitative Real-Time PCR Analysis

The RNA-seq analysis was performed by Biomarker Technologies Co., Ltd. (Beijing, China). Total RNA was isolated from broccoli seedlings using the RNA Pure kit for plants (Vazyme, Beijing, China). The PCR primers for 14 glucoraphanin biosynthesis-related genes were designed using Primer 5.0. A quantitative real-time PCR (qRT-PCR) analysis was conducted using the SYBR Premix Ex-Taq™ system (Q711, Vazyme). The reaction volume (10 μL) consisted of 1 μL template cDNA, 5 μL TB Green, 3.2 μL RNase-free water, 400 nM forward primer, and 400 nM reverse primer. The PCR program was as follows: 95 °C for 30 s; 40 cycles of 95 °C for 5 s and 60 °C for 30 s. An actin gene was used as the reference gene. Details regarding the primers designed for this study are provided in Table 1.

### 2.7. Statistical Analysis

All data are herein presented as the mean ± standard deviation (SD) of six biological replicates (*n* = 6). Statistical analyses were performed using the SPSS 25.0 software (IBM, Armonk, NY, USA). Data were analyzed using Tukey’s test. The threshold for determining significant differences was *p* < 0.05.

## 3. Results

### 3.1. Effects of Broccoli Seedling Age on Glucosinolate Content

**Figure 1:**
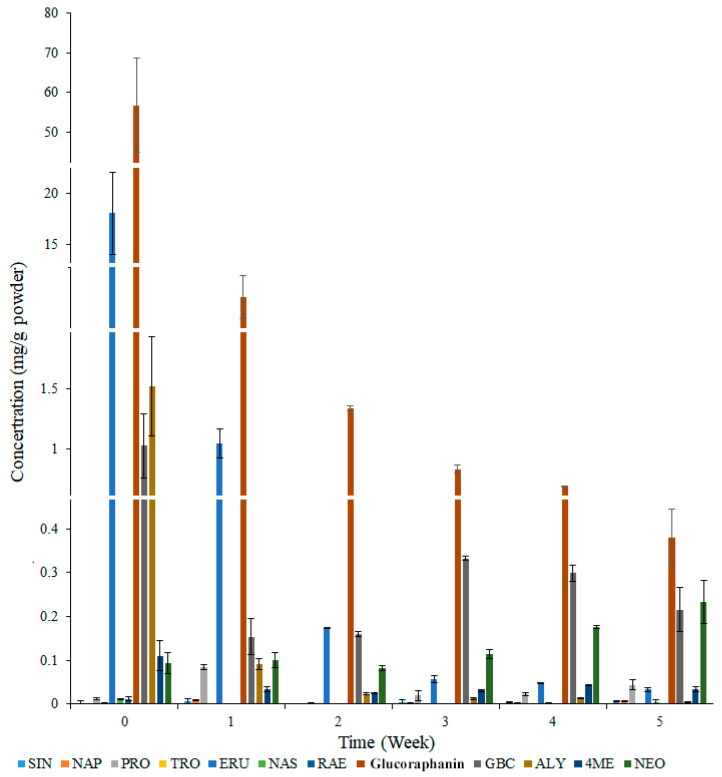
The concentration of varied glucosinolates during broccoli seeding. SIN, Sinigrin; NAP, Gluconapin; PRO, Progoitrin; TRO, Glucotropaeolin; ERU, Glucoerucin; NAS, Gluconasturtiin; RAE, Glucoraphenin; GBC, Glucobrassicin; ALY, Glucoalyssin; 4ME, 4-Methoxyglucobrassicin; NEO, Neoglucobrassicin.

Changes in the concentrations of the main chemical compounds in developing broccoli seedlings are presented in Figure 1. Among the analyzed glucosinolate compounds, glucoraphanin was the most abundant in broccoli seeds and seedlings. Compared with the corresponding levels in the W0 group, the glucoraphanin, ERU, and ALY concentrations decreased in the W5 group. There were no significant changes in the SIN, NAP, TRO, NAS, and RAE concentrations during the 5-week culture period. The PRO concentration increased in the W1 group, but decreased in the W2 group. Additionally, the PRO concentration tended to increase between weeks 2 and 5. There were no obvious changes in the NEO concentration during the first 3 weeks, but the NEO concentration increased from week 3 to week 5. The 4ME concentration demonstrated a decreasing trend during the culture period.

### 3.2. Effects of Broccoli Seedling Age on Glucoraphanin Content and the Broccoli Seedling Index

**Figure 2:**
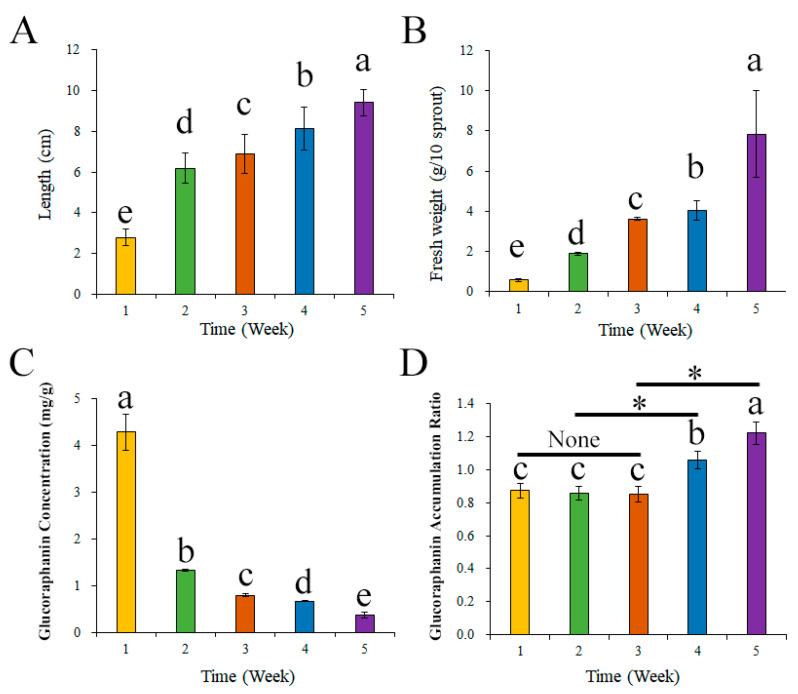
Broccoli seeding index and glucoraphanin content during growth stage. (**A**) Broccoli seeding length, (**B**) fresh seeding weight, (**C**) glucoraphanin content, and (**D**) glucoraphanin accumulation ratio. Different lowercase alphabet and * means significant difference at *p* < 0.05.

Figure 2 presents the changes in the glucoraphanin content and broccoli seedling status over time. The broccoli seedling length and fresh weight increased significantly after week 2 (*p* < 0.05) (Figure 2A,B). The glucoraphanin concentration was significantly higher for the W1 group than for the W2–W5 groups (*p* < 0.05) (Figure 2C). As shown in Figure 2D, the glucoraphanin accumulation ratio increased during the culture period and was significantly higher at weeks 4 and 5 than at week 1.

### 3.3. Effects of Broccoli Seedling Age on the Contents of Key Glucoraphanin Synthesis-Related Compounds and Molecular Metabolism

**Figure 3:**
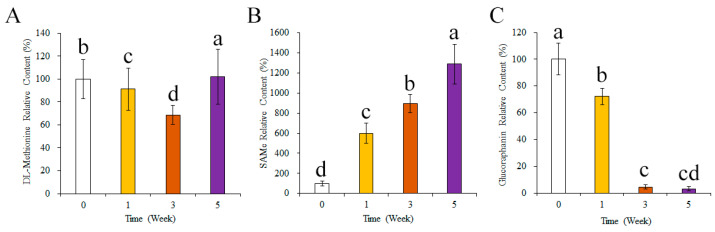
Contents of key substances in glucoraphanin synthesis during broccoli seeding. (**A**) relative content of DL-methionine, (**B**) relative content of SAMe, (**C**) relative content of glucoraphanin. SAMe, S-adenosyl methionine. Different letters mean significant difference at *p* < 0.05.

The changes in the contents of key compounds related to glucoraphanin synthesis during the broccoli seedling culture period are presented in Figure 3. The relative DL-methionine content was significantly lower in the W3 group than in the W0 group. However, the relative DL-methionine content increased significantly between weeks 3 and 5 (*p* < 0.05). The relative SAMe content increased significantly over time (W0 to W5) (*p* < 0.05). The relative glucoraphanin content was significantly lower in the developing broccoli seedlings than in the seeds. Notably, there was a significant decrease in the relative glucoraphanin content in the broccoli seedlings between weeks 1 and 3 (*p* < 0.05).

### 3.4. Effects of Broccoli Seedling Age on the Number of Differentially Expressed Genes

**Figure 4:**
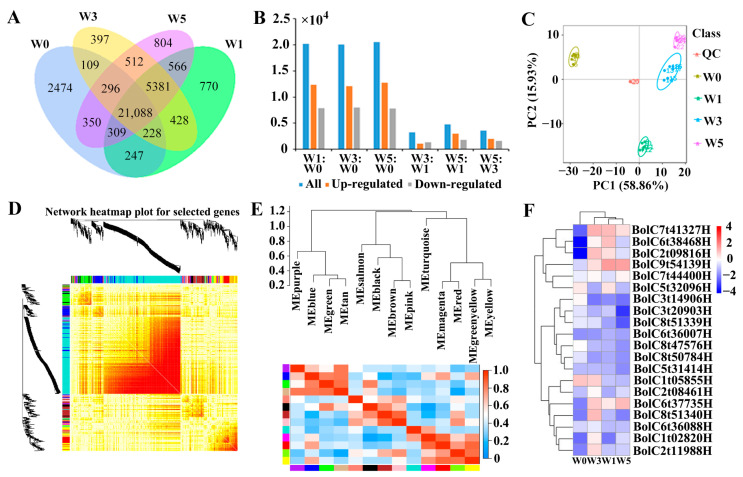
The number of different genes expressed during broccoli seeding time. (**A**) Number of genes expressed, (**B**) number of significantly different genes, (**C**) distribution of main different genes expressed, (**D**,**E**) dendrogram showing co-expression modules identified based on DEGs with RNA-seq with different colors, and (**F**) essential DEGs detected among the developmental periods of broccoli seedlings with RNA-seq.

The number of DEGs gradually changed as the broccoli seedlings developed (Figure 4). Many genes were expressed in broccoli seedlings at multiple time points or at specific time points (Figure 4A). The comparison with the W0 group revealed 20,206 DEGs in the W1 group (12,324 upregulated genes and 7882 downregulated genes), 20,070 DEGs in the W3 group (12,110 upregulated genes and 7960 downregulated genes), and 20,555 DEGs in the W5 group (12,772 upregulated genes and 7783 downregulated genes) (Figure 4B). The comparison with the W1 group detected 3218 DEGs in the W3 group (1889 upregulated genes and 1329 downregulated genes) and 4771 DEGs in the W5 group (2961 upregulated genes and 1810 downregulated genes) (Figure 4B). The comparison with the W3 group identified 3579 DEGs in the W5 group (2010 upregulated genes and 1569 downregulated genes) (Figure 4B). Moreover, the total number of expressed genes differed significantly among the examined time points (Figure 4C).

We found that there were five modules corresponding to aliphatic glucosinolate metabolism based on WGCNA analysis (Figure 4D). The largest module was marked MEpink (Figure 4E), while the smallest module was marked MEblue. All modules are shown in Figure 4E. Moreover, we detected 20 essential DEGs and their express pattern (Figure 4F), which provided new insight into understanding the developmental mechanism of broccoli breeding.

### 3.5. Effects of Broccoli Seedling Age on the Expression of Glucoraphanin Synthesis-Related Genes

**Figure 5:**
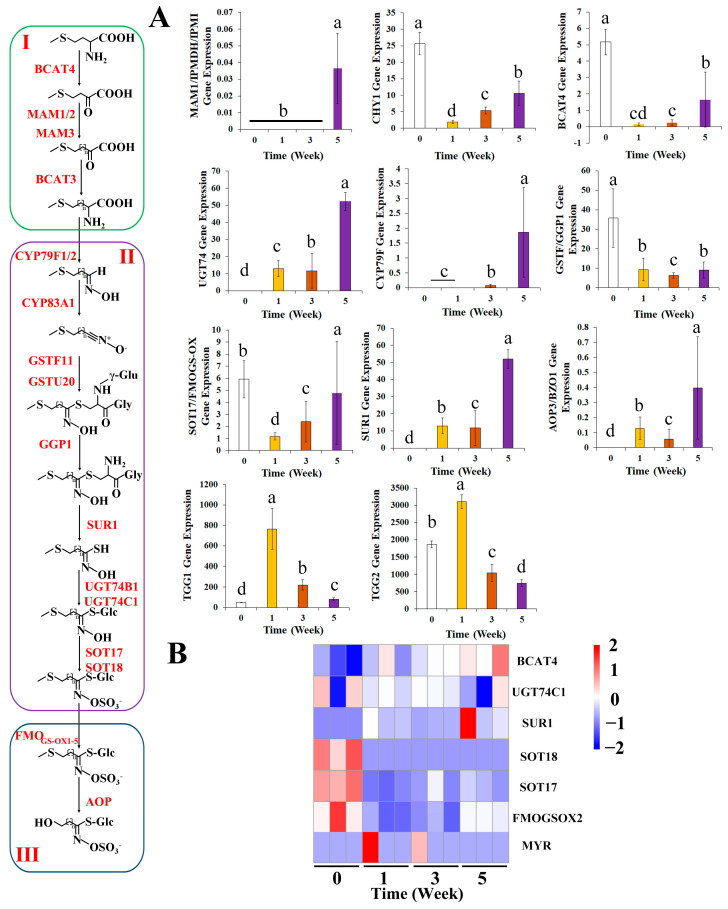
Main genes expressed in glucoraphanin biosynthesis during broccoli seeding time. (**A**) main significantly different genes and (**B**) heatmap of differentially expressed genes. Different alphabet means significant difference at *p* < 0.05.

*MAM1*, *IPMDH*, and *IPM1* expression levels were significantly higher in the W5 group than in the W0, W1, and W3 groups. In addition, *BCAT4*, *GSTF*, and *GCP1* expression levels decreased significantly after germination (Figure 5, *p* < 0.05). Conversely, *SUR1* was more highly expressed in the seedlings than in the seeds, with a significantly higher expression level in the W5 group than in the W1 and W3 groups (Figure 5A, *p* < 0.05). *SOT17* and *FMOGS-OXs* expression levels were significantly lower in the W1 group than in the W0 group (Figure 5, *p* < 0.05). A comparison of the *CYP79F1* expression levels indicated that this gene was significantly more highly expressed in the W3 group than in the W0 group and significantly more highly expressed in the W5 group than in the W1 and W3 groups (Figure 5A, *p* < 0.05). The *TGG1* and *TGG2* expression levels increased significantly in the W1 group, but decreased significantly in the W3 and W5 groups (Figure 5A, *p* < 0.05).

The *SOT18* and *SOT19* genes showed a high expression level at 0 weeks, while there were no expression profiles detected over 1 to 5 weeks, which indicated that both genes might have special expression patterns in broccoli organs and tissues. The *FMOGS-OX2* and *FMOGS-OX5* genes also presented higher expression levels in sprouts, and their content decreased gradually after 1 week, which was consistent with the change in glucoraphanin contents. Therefore, this finding might indicate that *FMOGS-OX5* genes played a positive role in regulating glucoraphanin generation.

### 3.6. Effects of Broccoli Seedling Age on the Relative Expression Levels of the Genes Related to Glucoraphanin Synthesis

**Figure 6:**
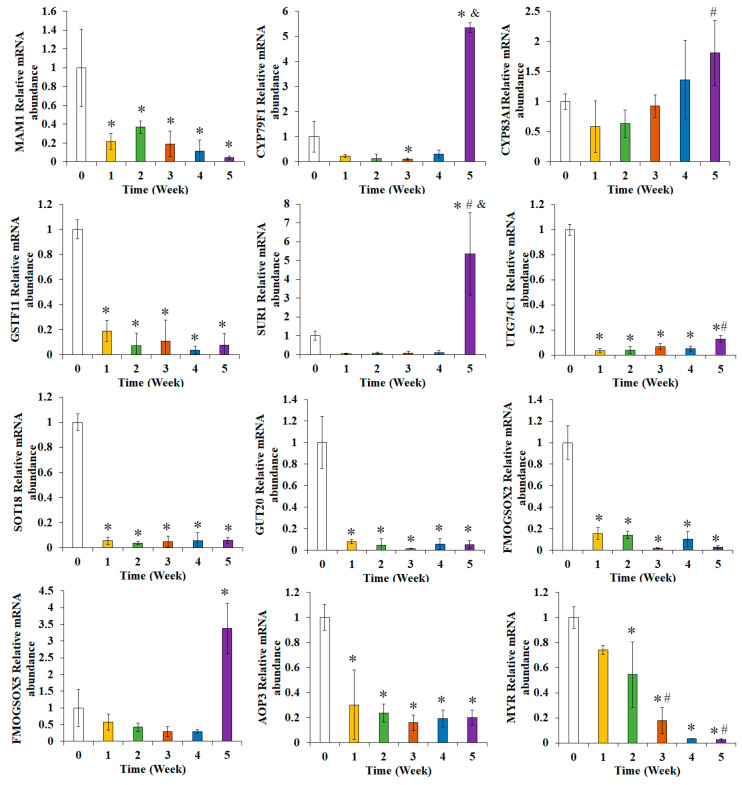
Relative abundance of mRNA for genes related to glucoraphanin biosynthesis and metabolism in broccoli seedlings. *, significant difference compared with the W0 group (*p* < 0.05); #, significant difference compared with the W1 group (*p* < 0.05); &, significant difference compared with the W3 group (*p* < 0.05).

The genes associated with glucoraphanin biosynthesis and metabolism were detected via RT-PCR technology (Figure 6). The relative abundance of mRNA for *MAM1*, *GSTF11*, *UTG74C1*, *SOT18*, *GOT20*, *FMOGS-OX2*, *FMOGS-OX5*, and *AOP3* decreased significantly in the broccoli seedlings (relative to the corresponding levels in the seeds) (*p* < 0.05). Additionally, the relative abundance of the *UTG74C1* mRNA was significantly higher in the W5 group than in the W1 group (*p* < 0.05). The relative abundance of the *CYP79F1* mRNA was significantly lower in the W3 group than in the W0 group. Furthermore, it was significantly higher in the W5 group than in the W0 and W3 groups (*p* < 0.05). Compared with the corresponding level in the samples at week 1, the relative abundance of the *CYP83A1* mRNA increased significantly after week 4 (*p* < 0.05). The *SUR1* expression level was significantly higher in the W5 group than in the W0, W1, and W3 groups (*p* < 0.05). The *FMOGS-OX5* expression level in the W5 group was significantly higher than that in the W0 group (*p* < 0.05). The MYR expression level in the seedlings decreased significantly after 2 weeks (relative to the corresponding level in the seeds). In addition, the *MYR* expression levels in the W3 and W5 groups were significantly lower than those in the W1 group (*p* < 0.05).

## 4. Discussion

Earlier research showed that glucoraphanin is an important functional glucosinolate among the glucosinolates commonly found in cruciferous plants [31]. Glucoraphanin metabolites can inhibit the growth of cancer cells and enhance the activity of prothyrotropin [32]. Because of the diversity in total glucosinolate content during broccoli growth, clarifying the glucoraphanin/total glucosinolate ratio in broccoli seeds and during the germination process of broccoli is necessary [7]. Nieto et al. reported that glucoraphanin is the main glucosinolate in broccoli [33], which is consistent with the glucoraphanin accumulation ratio calculated in the current study (Figure 1). In addition, Li et al. observed that in broccoli, the glucoraphanin content is higher in the seeds than in the other organs [34]. Unfortunately, humans cannot obtain glucoraphanin directly from broccoli seeds. Instead, humans often obtain glucoraphanin from broccoli sprouts or mature broccoli plants, which necessitates broccoli cultivation [35]. Researchers have also determined that glucoraphanin concentrations are significantly lower in broccoli seedlings than in broccoli seeds [36]. There has been considerable interest in increasing the glucoraphanin content of broccoli sprouts during a 1-week growth period, but there has been relatively little research on whether other time periods (e.g., seedling development stage) may be better in regulating the glucoraphanin content of broccoli [37,38]. In the current research, the glucoraphanin accumulation ratio was higher in broccoli seedlings (after 4 weeks) than in broccoli seeds (Figure 2). Although the broccoli seedling length and fresh weight increased significantly, the glucoraphanin concentration in broccoli seedlings decreased significantly. Thus, the increase in the glucoraphanin accumulation ratio was not due to an increase in the seedling length and fresh weight. It is also possible that glucoraphanin synthesis was initiated at approximately 4 weeks after germination [38]. In order to clarify the mechanism underlying the increase in the glucoraphanin accumulation ratio, the key components of the glucoraphanin biosynthesis pathway were analyzed.

Similar trends were observed for the changes in the glucoraphanin concentration and relative content (Figure 2 and Figure 3). In addition, glucoraphanin is synthesized from DL-methionine, but DL-methionine may also be converted to SAMe. Hence, the DL-methionine content is an important factor influencing glucoraphanin synthesis [39]. In the present study, the relative DL-methionine content in broccoli seedlings increased significantly after 3 weeks and was higher than the corresponding content in broccoli seeds at week 5 (Figure 3). The relative SAMe content also increased, possibly because of the increase in the relative DL-methionine content. Furthermore, similar trends were reflected in the glucoraphanin accumulation ratio and the relative DL-methionine or SAMe content changes (Figure 2 and Figure 3). To further clarify the mechanism mediating the increase in the glucoraphanin accumulation ratio, the expression levels of the genes encoding enzymes related to glucoraphanin synthesis were analyzed. A previous investigation indicated the enzyme content may affect how efficiently glucoraphanin is synthesized [25].

Zhao et al. detected significant changes in the expression of glucoraphanin synthesis-related genes during the germination period [40]. Of the examined genes in the present study, approximately 60% had an upregulated expression level; these genes included the main genes encoding glucoraphanin biosynthesis-related enzymes (Figure 4). Wang et al. revealed that BCAT4 can convert DL-methionine to 2-oxoacid and increase the glucoraphanin content [41]. Other researchers reported that *CHY1* can promote glucoraphanin synthesis [16]. In the current study, *BCAT4* and *CHY1* expression levels increased at week 5 (Figure 5), which may help to explain the increase in the relative SAMe content. Borpatragohain et al. showed that *CYP79F1*, *SUR1*, and *UGT74* also positively affect glucoraphanin synthesis [42]. We observed that *CYP79 F1*, *SUR1*, and *UGT74* expression levels increased at week 5 (i.e., only in broccoli seedlings) (Figure 5). However, many of the glucoraphanin synthesis-related genes were not precisely detected.

The changes in the expression of glucoraphanin synthesis-related genes were more comprehensively explored by completing RT-PCR analyses. The expression of *CYP79F1*, *CYP83A1*, and *FMOGS-OX5* may enhance the conversion of precursor amino acids to activated compounds, which are subsequently used for secondary modifications [43]. The observed increase in the glucoraphanin accumulation ratio may have been caused by changes in the expression of *CYP79F1*, *CYP83A1*, and *FMOGS-OX5* (Figure 6). However, glucoraphanin synthesis is reportedly accompanied by decreases in the extent of glucoraphanin degradation and transformation [44]. In the present study, the AOP3 and MYR expression levels decreased significantly at week 2. *TGG1*, *TGG2*, and *MYR* expression levels decreased to very low levels (Figure 5 and Figure 6). Thus, the increase in the glucoraphanin accumulation ratio may have been the result of increased glucoraphanin synthesis and decreased glucoraphanin degradation.

## 5. Conclusions

As the absolute glucoraphanin content of broccoli sprouts was more significant than glucoraphanin concentration in broccoli seedlings during 5 weeks, it is necessary to clarify the mechanism of glucoraphanin accumulation during broccoli germination. Our results suggest that the increase in absolute glucoraphanin content in broccoli seedlings might be due to an increase in glucoraphanin biosynthesis and a decrease in glucoraphanin conversion at week 3, guiding healthy diet recommendations for humans and the industrial production of broccoli. Additionally, a total of 20 potential regulation genes related to the development of broccoli seedlings have been provided for future verification. Therefore, this research proposes that by upregulating *CYP79F1* and *FMOGS-OX5* genes, higher broccoli seedling glucoraphanin contents may be obtained after 3 weeks.

## Figures and Tables

**Table 1:** Genes related to glucoraphanin synthesis and their qRT-PCR primers.

Gene Name	Sequence (5′-3′)	Expected Size (bp)
*UTG74C1*	Forward: CCTGACCGATTTCATCTCTAGTGC	24
	Reverse: TGGCTATGTCCAATGCAAAGGG	22
*GSTF11*	Forward: CTTTGGAGGGACGAGCCATT	20
	Reverse: TGTGAATTCATCACCGCCCA	20
*FMO-GS-OX2*	Forward: GACCGTGGTTACGGGAGACTTG	22
	Reverse: GTAGCCATTGTATAACAAGCAACCC	25
*FMO-GS-OX5*	Forward: CGAACATGTCTTTCCGCCTG	20
	Reverse: TCTTAGGTTGCCCGAAAGCC	20
*AOP3*	Forward: TTGATGCGGAGTTGGGCTTA	20
	Reverse: CTCGGTGATACGGTGAAGGG	20
*MYR*	Forward: GATGGGCGAACTCAATGCTAC	21
	Reverse: CACTCCCCTACTCACCTTTCCTT	23
*MAM1*	Forward: AAATTCTGGCATTGCTCGTGG	21
	Reverse: ATCACCAGATTCACCGCACG	20
*GSTU20*	Forward: GCTAGAGTTGCGTTGCGAGA	20
	Reverse: CCCGTAAGGATCGGAAGGGA	20
*CYP83A1*	Forward: TTCATATCCTACGGCAGGCG	20
	Reverse: TTATCCGCGGCCTTGTTGAT	20
*SOT18*	Forward: CAAGGCTACGATCACGACCA	20
	Reverse: GACGGTAGCCACCAGTAACC	20
*SUR1*	Forward: CCAAGCCGTGTGATTGTACG	20
	Reverse: TTGTGGCCCTAGACACTGGA	20
*CYP79F1*	Forward: ATCAATCAGTTTGCTTGGCCG	21
	Reverse: CCTTTCAAGGCGATGTCGAT	20
*Actin*	Forward: CTGTTCCAATCTACGAGGGTTTC	23
	Reverse: GCTCGGCTGTGGTGGTGAA	19

## Data Availability

Data are contained within the article.
