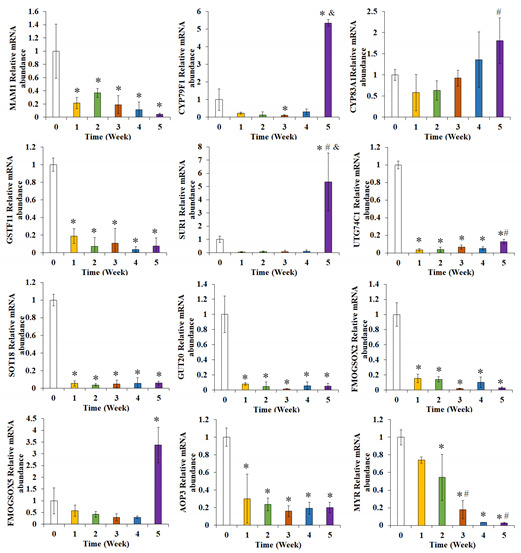# Correction: Liu et al. Glucoraphanin Accumulation via Glucoraphanin Synthesis Promotion during Broccoli Germination. *Foods* 2024, *13*, 41

**DOI:** 10.3390/foods13152466

**Published:** 2024-08-05

**Authors:** Guangmin Liu, Hongju He, Pengjie Wang, Xirui Zhao, Fazheng Ren

**Affiliations:** 1 Key Laboratory of Functional Dairy, Ministry of Education, Department of Nutrition and Health, China Agricultural University, Beijing 100083, China; 2 Institute of Agri-Food Processing and Nutrition, Beijing Academy of Agricultural and Forestry Sciences, Beijing 100097, China

In the original publication [1], there was a mistake in Figure 6 as published. The result of “AOP3 relative mRNA abundance” has a repeated occurrence in Figure 6. The 12th figure of Figure 6 should be the result of “MYR relative mRNA abundance”. The corrected Figure 6 appears below. The authors state that the scientific conclusions are unaffected. This correction was approved by the Academic Editor. The original publication has also been updated.